# Innovative Membrane Purification of Plant-Based Lipase from Flaxseed Using Graphene Oxide

**DOI:** 10.1021/acs.jafc.5c12394

**Published:** 2025-12-26

**Authors:** Nicole Novelli do Nascimento, Paulo Waldir Tardioli, Rosângela Bergamasco, Angélica Marquetotti Salcedo Vieira

**Affiliations:** † Postgraduate Program in Food Science, Centre of Agrarian Sciences, 42487 State University of Maringá, Av. Colombo, 5790, CEP 87020-900 Maringá, PR, Brazil; ‡ Postgraduate Program in Chemical Engineering, Graduate Program in Chemical Engineering, Department of Chemical Engineering, 67828 Federal University of São Carlos, Rodovia Washington Luís, km 235, SP-310, CEP 13565-905 São Carlos, SP, Brazil; § Department of Chemical Engineering, State University of Maringá, Av. Colombo, 5790, CEP 87020-900 Maringá, PR, Brazil; ∥ Department of Food Engineering, State University of Maringá, Av. Colombo, 5790, CEP 87020-900 Maringá, PR, Brazil

**Keywords:** lipase, flaxseed byproduct, biocatalytic processes, modified membrane technology, alternative purification

## Abstract

Plant-derived lipases offer a cost-effective and sustainable alternative to microbial and animal enzymes for industrial use. Flaxseed is a promising source due to its high lipase activity during germination, although efficient extraction of the active enzyme remains challenging. This study evaluates optimized extraction and purification strategies for lipase from germinated, defatted flaxseed. Maximum specific activity was observed after 4 h of germination. Purification using a membrane modified with polyethylenimine (PEI) and graphene oxide (GO) nanoparticles functionalized with tannic acid (TA) significantly enhanced performance, increasing specific activity by approximately 11.6-fold. Membrane characterization showed reduced pore size and improved antifouling behavior, allowing reuse for at least two cycles with a 74.38% flow recovery. The purified lipase demonstrated thermal stability between 25 and 60 °C. Overall, the proposed membrane-based approach enables the efficient production of high-activity flaxseed lipase, reinforcing its potential as a sustainable and economically viable industrial biocatalyst.

## Graphical abstract

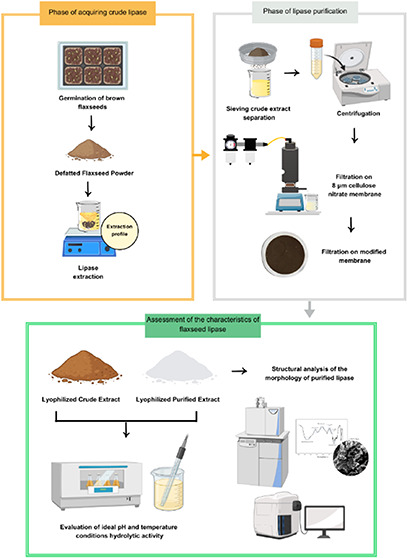

## 1 Introduction

Industrial interest in biocatalytic processes has increased in recent years, particularly driven by the demand for sustainable and eco-friendly processes.^1,2^ Within this context, the application of enzymes, especially lipases, has gained attention due to their high catalytic efficiency and versatility, with notable applications in diverse industries.^3–5^ However, the large-scale use of plant-derived lipases still faces challenges, primarily related to the purification process, which can lead to a loss of enzymatic activity and increased production costs.

Lipases (E.C. 3.1.1.3) are versatile enzymes widely used in biotechnology due to their ability to catalyze reactions such as hydrolysis, esterification, and transesterification at the oil–water interface under mild conditions.^6,7^ Their catalytic promiscuity allows their application in the food industry, where lipases are essential for the modification of lipids and the production of omega-3 fatty acids.^8–10^

Although microbial lipases are widely used, their high production costs have driven interest in plant-based alternatives that are more accessible and cost-effective. Despite their favorable properties, such as activity across broad pH (4–9) and temperature (37–80 °C) ranges, plant lipases receive limited attention due to challenges in extraction and purification, which often lead to enzyme activity loss. However, their tolerance to organic solvents and substrate specificity make them promising biocatalysts. Oleaginous plants such as flaxseed are particularly notable sources, with potential applications in biodiesel and lipid production. Still, scaling up remains a challenge due to low extraction yields from plant material.^11–13^

The extraction of lipases from plants is challenging due to their localization in lipid-rich tissues, where they support energy mobilization during germination. In oilseeds, triacylglycerols stored in oil bodies serve as energy reserves, and lipolytic enzymes like lipases are only expressed in significant amounts during germination.^14,15^ As a result, germination directly influences lipase activity, making it essential to obtain crude extracts from germinated seeds to assess enzymatic performance.^8,16^ In nongerminated seeds, lipolytic activity is typically absent, and postgermination, lipases may localize in lipid bodies or other cellular compartments.^17^

Purifying the crude enzymatic extract is a crucial step for enhancing enzyme activity and operational stability. Different methods have been developed for the purification of lipases, ranging from conventional techniques such as precipitation, dialysis, and chromatography to more advanced approaches involving affinity and immunoaffinity systems. Traditional purification processes often require multiple steps and can be time-consuming, costly, and lead to partial enzyme inactivation.^10,18–20^ In recent years, membrane-based separation technologies have emerged as an efficient and scalable alternative for lipase purification. Compared with traditional chromatographic processes, membrane systems offer continuous operation, lower solvent consumption, and reduce energy demand. For instance, ultrafiltration membranes have achieved lipase recovery yields above 85% with permeate fluxes exceeding 120 L·m^–2^·h^–1^ under mild operating conditions, while maintaining enzyme activity and structural integrity. The application of surface-modified membranes (MM), particularly those functionalized with hydrophilic or charged layers, further enhances the selectivity and antifouling resistance, leading to improved stability and reproducibility during repeated use. Such modified systems can increase the purification yield by 15–25% and extend membrane lifespan compared to unmodified counterparts. Therefore, membrane separation—especially when using surface-engineered materials—represents a promising, sustainable, and high-performance alternative for lipase purification in both laboratory-scale and industrial bioprocesses.^21–26^

In this context, poly­(ether sulfone) (PES) microfiltration (MF) membranes modified with polyethylenimine (PEI) and graphene oxide (GO) nanoparticles functionalized with tannic acid (TA) have been explored as an effective purification strategy. PES membranes are generally used in the coating process due to the ease of surface modification. These are hydrophobic membranes that are more prone to fouling, so it is necessary to hydrophilize the membrane surface to promote the antifouling property during filtration processes.^23,24^ The incorporation of hydrophilic agents, such as PEI, helps to reduce fouling, increasing membrane hydrophilicity.^25–27^ There are also GO nanoparticles, which have functional groups containing oxygen that can attribute hydrophilicity and have excellent film-forming capacity in PES membranes. Furthermore, they provide antifouling, mechanical, and chemical properties.^28,29^ These nanoparticles can be functionalized to reduce membrane porosity and improve selectivity, as seen in the case of GO polymerization by tannic acid.^30,31^

In the present study, this modified membrane system was applied as an innovative alternative for purifying the crude lipase extract obtained from germinated and defatted flaxseed (*Linum usitatissimum* L.).^32,33^ Flaxseed is rich in triglycerides, and its oilseeds have a high lipase content, being an alternative source of vegetable lipase.^34,35^ It thrives in temperate climates, with global production reaching 3.3 million tons in 2021, led by Russia, Kazakhstan and Canada.^36,37^

The purpose of this study was to purify the enzymatic extract using PES membranes modified with PEI and GO functionalized with tannic acid and to evaluate the efficiency of the separation process, the membrane’s performance, and the enzymatic activity of the purified lipase. This approach represents a cost-effective and efficient alternative to conventional purification techniques and supports the potential of plant-based lipases for industrial-scale applications.

## 2 Materials and Methods

### 2.1 Chemicals

Brown flaxseeds (Dona Nena and Vitao trademarks) were obtained from a local supermarket in Maringá, PR, Brazil, and stored in a freezer until use. Olive oil (Andorinha trademark) was purchased from a local supermarket (Maringá, PR, Brazil). Gum Arabic powder and tannic acid were purchased from Synth (Diadema, SP, Brazil). Polyethylenimine (PEI, *M*_w_ = 1300, 50% in H_2_O), GO solution (2 mg/mL), bovine serum albumin, and polyacrylamide were purchased from Sigma-Aldrich (Saint Louis, MO). Brilliant Coomassie Blue was purchased from Dinâmica (São Paulo, SP, Brazil). Commercial PES microfiltration (MF) membrane (47 mm diameter, thickness 150 and 0.20 μm average pore size) and cellulose nitrate filters (47 mm diameter, 8 μm average pore size) were obtained from Sartorius (Goettingen, Lower Saxony, Germany). All other chemicals were of analytical grade and used as received.

### 2.2 Flaxseed Germination

Germination of flaxseed was performed based on refs 38–40 with adaptations. First, the seeds were washed with 70% ethyl alcohol for 3 min and then with deionized water. The seeds were soaked in water at a ratio of 1:6 (seed weight in grams to water volume in milliliters) and spread in a thin layer in plastic trays. The aim of this procedure was to accelerate the metabolic and cellular activities and, consequently, to increase the lipolytic activity.^13^ Germination was carried out for 10 days at room temperature (20–30 °C) protected from light. The seeds were washed twice a day with 70% ethyl alcohol and deionized water to prevent microbial growth.

### 2.3 Seed Defatting

After the germination stage, seed size was reduced according to refs 32,41 with adaptations, aiming to increase the solid–liquid contact surface, facilitating the extraction of lipase that is in subcellular compartments.^42^ Before selecting acetone as the solvent for the defatting process, preliminary tests were performed using hexane and ethanol to evaluate their effectiveness in oil removal. Based on these trials, acetone was chosen as the most efficient solvent, and the extraction time was established according to studies on linseed oil extraction.^36,43^

The germinated seeds were dried (40 °C for 24 h) in a Sterilifer microprocessed stove (São Caetano do Sul, SP, Brazil) since the presence of water can reduce the yield of oil extraction with organic solvents (seed degreasing step).^43,44^ They were weighed and crushed with the aid of a blender. After that, cold acetone (4 °C) was used to extract the flaxseed oil at a ratio of 1:10 m/v (flaxseed powder/acetone volume) for 4 h under magnetic stirring and temperature control (max. 8 °C) in containers sealed and protected from light. The extraction procedure was carried out twice to increase the oil extraction efficiency. The flaxseed oil was recovered by evaporation under vacuum in a rotary evaporator (TE-211, Tecnal, Piracicaba, SP, Brazil) at 40 °C until complete evaporation of the acetone.

The defatted flaxseed powder underwent recovery through a 150 mesh sieve, followed by washing with 95% ethyl alcohol and deionized water. Subsequently, it was stored in a refrigerator for later utilization in lipase extraction. However, before employing it for this purpose, the enzymatic activity of the defatted flaxseed powder was assessed in accordance with the procedure outlined in Section 2.4.1.

### 2.4 Lipase Extraction

The defatted flaxseed powder was used to extract proteins following the methodology described by Mehdi et al.^45^ with adaptations. This step was performed using 100 mM sodium phosphate buffer at pH 7.0 with a powder mass/buffer volume ratio of 1:30 for up to 24 h under magnetic agitation and protected from light in an ice bath. The extraction of lipase from flaxseed is commonly carried out at pH 7 because this value is close to the enzyme’s physiological pH, helping to preserve its native conformation and catalytic activity while minimizing the risk of denaturation or loss of functionality during the extraction process.^45^

Samples of filtrate (solid-free liquid phase) and whole suspension (liquid plus powder) were collected at intervals from 15 min to 24 h to evaluate the best time to extract proteins with higher enzyme activity.

The protein crude extract was stored in a refrigerator for further use, and the solid residue was dried and stored in a freezer for future evaluation.

The lipase productivity from flaxseed was calculated as the ratio of the lipolytic activity of the crude extract to the total mass of dry seed used in the process expressed in units per gram of dry weight.

#### 2.4.1 Enzymatic Activity

Olive oil hydrolysis was performed according to the methodology described in ref 46 with adaptations. A mixture of 10 mL of an emulsion composed of 2.5 mL of olive oil, 0.1 g of gum arabic, 2.5 mL of water, and 5 mL of 100 mM sodium phosphate buffer, pH 7.0, was prepared. The hydrolysis reaction took place for 5 min after adding the enzymatic solution (2 mL) at 37 °C in a water bath (Dubnoff type, QEC brand, model 226M2) with stirring (230 rpm). After this time, 10 mL of a 95% ethanol solution was added. The released fatty acids were titrated with a 0.1 M NaOH solution by using phenolphthalein as an indicator. A unit of activity (U) was defined as the amount of enzyme that releases 1 μmol of fatty acid per minute under the specified reaction conditions and was expressed as U per milliliter of enzyme solutions. The enzymatic activity was calculated according to eq 1, and specific activity (Li et al.) was expressed as U per mg of protein (eq 2).

1 $$\mathrm{enzymatic}\;\mathrm{ativity}\left(\frac{\mu \mathrm{mol}}{\min\mathrm{mL}\;\mathrm{or}\;\mathrm{mg}}\right)=\frac{\left(V_{A}-V_{B}\right)\times M\times 1000}{t\times E}$$

where *E* is the amount of enzyme used in the assay (mL for the soluble enzyme, and mg for the immobilized enzyme); *M* is the molarity of the NaOH solution; *t* is the reaction time (min); *V*_A_ is the volume of NaOH used in the titration of the sample (mL); and *V*_B_ is the volume of NaOH spent in the blank titration (mL). A unit of activity (U) is defined as the amount of enzyme that releases 1 μmol of fatty acid per minute under the reaction conditions. Specific activity (Li et al.) was calculated according to eq 3

2 $$\mathrm{specific}\;\mathrm{activity}\left(\frac{U}{\mathrm{mg}}\right)=\frac{\mathrm{enzyme}\;\mathrm{activity}}{\mathrm{mass}\;\mathrm{of}\;\mathrm{total}\;\mathrm{proteins}}$$

#### 2.4.2 Protein Content

Protein content was quantified by the Bradford method^47^ using bovine serum albumin as a standard protein to construct the standard curve. Sample absorbances were measured at 595 nm.

#### 2.4.3 Electrophoresis

SDS-PAGE electrophoresis was performed under denaturing conditions using a polyacrylamide gel system, comprising a 3.75% stacking gel and a 12.5% separating gel, as described by Laemmli.^48^ Samples were prepared by diluting the crude extract 2-fold and the purified extract 5-fold, ensuring both contained equivalent protein concentrations. Molecular weight standards (10–250 kDa, Bio-Rad) were employed to estimate the molecular weight of the lipase by comparing its electrophoretic mobility to that of the standard markers. Protein bands were visualized using Coomassie Brilliant Blue R-250 staining.

### 2.5 Membrane Modification

Graphene oxide was prepared based on the Hummers’ method adapted from ref 49. From a calibration curve prepared spectrophotometrically (600 nm) with a commercial suspension of GO, the concentration of the suspension of GO was obtained. In the suspension of 1.00 mg of GO in 30 mL of deionized water, 4 mg of tannic acid (TA) with pH adjusted to 8.5 with NaOH and HCl solutions was added for functionalization to occur (TA-GO).^50^ The methodology based on the modification of MF membranes through the coating technique was as in refs 50–52 . The PES membranes were immersed in a Petri dish containing deionized water for 5 min with manual shaking to remove the chemicals added by the manufacturer. Subsequently, they were transferred to the same membrane module described above. The modifier solutions were filtered with atmospheric pressure in the following order: 500 μL of PEI diluted in 20 mL of deionized water, 30 mL of a previously prepared aqueous solution of TA-GO, and another layer of 500 μL of PEI diluted in 20 mL of deionized water. After the modifications, all filtrations were carried out immediately on the same membrane module. All filtrations of the modified membranes were carried out at room temperature (25 °C).

### 2.6 Purification Steps

Following the lipase extraction process, the result is a crude enzymatic extract. To enhance the enzymatic activity, further purification steps are required. For this, sieving, centrifugation, and filtration were carried out on commercial membranes with and without modification.

After the lipase extraction time, the crude extract was separated from the residue by decantation, followed by separation on a 106 mm Bertel (Caieiras, SP, Brazil) sieve for particle size analysis. After these techniques, centrifugation was performed to remove solid particles that could interfere with the filtration step, clogging the membranes and reducing purification efficiency. The crude extract was centrifuged in three cycles of 2000 rpm for 10 min in a Corning LSE Compact Centrifuge (New York), resulting in a preclarified extract. An aliquot was collected from each step for later analysis.

#### 2.6.1 Purification of the Preclarified Extract Using Membranes

The 8 μm cellulose nitrate membrane (CNM) was immersed in a Petri dish containing deionized water for 5 min with manual shaking to remove the chemicals added by the manufacturer. Subsequently, the membrane was transferred to a bench-scale membrane module (capacity of 250 mL and filtration area of 11.34 cm^2^, PAM-Selective Membranes, Rio de Janeiro, RJ, Brazil) coupled to a pressurized compressed air system and flow perpendicular to the membrane. After filtration of the entire preclarified crude extract was obtained the clarified extract.

The clarified extract was then subjected to filtration using the surface-modified PES membrane, thus obtaining the purified extract. Initially, the permeability of the modified membranes was determined, and then filtration tests were performed, as described below.

Initially, the membranes were compacted for 15 min with deionized water under atmospheric pressure and 5 min under a pressure of 4 bar. Then, the hydraulic permeability of the modified membrane was performed, which was evaluated by the flow of water through the membrane (200 mL of water for 1 h) under different pressures (2, 4, 5, and 6 bar).

The initial water flux (*J*_0_) through the modified membrane was evaluated under a pressure of 4 bar and a volume of 200 mL for 1 h. Aliquots were collected at 10 min intervals during filtration. Then, the flow of the feeding solution (*J*_1_) was evaluated until stabilization (6 bar, 50 mL for 2h30) and, finally, water was used to evaluate the final flow (*J*_2_) for 1 h, under the same conditions described for *J*_0_. The experiments were performed in triplicate, and the results are expressed as the mean ± standard deviation. The fluxes (*J*_P_) were calculated according to eq 3

$$J_{P}=m/\rho \cdot t\cdot A$$ 3

where *J*_P_ represents the permeate flux (*J*_0_, *J*_1_, and *J*_2_) (L·h^–1^·m^–2^); *m* is the mass of the filtered solution (kg); ρ is the specific mass of water or phosphate buffer solution (kg·m^–3^); *t* is the time (h); and *A* is the effective membrane area (m^2^).

#### 2.6.2 Reuse of the Modified Membrane

The modified membrane (MM) was evaluated for the possibility of its reuse by filtering the feed solution in multiple cycles. For this, between each cycle and before the final flow of water, 20 mL of 50 mM phosphate buffer solution pH 7.0 was added to the module and left to stand at atmospheric pressure for 15 min (MM_R_). Then, this solution was removed, and the same amount was added again, but the buffer was filtered through the membrane at 4 bar for 15 min (MM_F_). The purification process involves transitioning from the crude extract to obtaining a purified enzyme extract through various sequential steps. Aliquots of these steps were collected for further analysis of protein content and enzymatic activity due to the possibility of recovering lipase that could be on the surface or pores of the membrane, and that could also eventually cause incrustations.^53,54^

To assess the antifouling effect of the modified membranes, the flux recovery rate (% FRR), total fouling (% *F*_T_), reversible fouling (% *F*_R_), and irreversible fouling (% *F*_I_) values were calculated using eqs 4–7, respectively.

4 $$\%\;FRR=J_{2}/J_{0}\times 100$$

5 $$\%\;F_{T}=\frac{J_{0}-J_{1}}{J_{0}}\times 100$$

6 $$\%\;F_{R}=\left(J_{2}-J_{1}\right)/J_{0}\times 100$$

7 $$\%\;F_{I}=\left(J_{0}-J_{2}\right)/J_{0}\times 100$$

### 2.7 Physicochemical Characterization of Flaxseed Lipase

The pH and temperature of maxima activities and thermal and pH stabilities were assessed for lyophilized crude and purified extract. The lyophilization was carried out in a lab lyophilizer (α model 2–4 LSCbasic, Christ, Osterode am Harz, Germany) at −50 °C with a vacuum of 0.04 mbar. All assays were carried out in a Shaker incubator (SL-222 model, Piracicaba, SP, Brazil) with constant stirring at 230 rpm.

#### 2.7.1 pH of Maximum Activity

Activities were measured at 37 °C in a pH range of 3–9. The emulsion of olive oil was prepared at the desirable pH, and the reaction was carried out as described in Section 2.4. Enzymatic activity at each pH value was calculated by eq 1.

The buffer solutions were prepared as follows: 50 mM sodium acetate (pH values of 3 and 5), 50 mM potassium biphthalate (pH 4), 50 mM sodium phosphate (pH values of 6 and 7), and Tris-HCl (pH values of 8 and 9).

#### 2.7.2 Temperature of Maximum Activity

Activities were measured at temperatures of 25, 37, 45, 50, and 60 °C and at the pH of maximum activity previously determined (Section 2.7.1) according to the enzymatic activity protocol (Section 2.4.1), using a mass of enzyme extract of ca. 30 mg. Enzymatic activity at each temperature was calculated by eq 1.

#### 2.7.3 pH Stability

The pH stability of the crude and purified enzyme extract was evaluated at different pH values. For all assays, ca. 100 mg of enzyme extract was added to 50 mL buffer solution and incubated at 37 °C. Samples of 2 mL were withdrawn at time intervals from 5 min to 120 h for measuring residual enzyme activity according to Section 2.4.1. Residual activities were calculated using eq 1.

#### 2.7.4 Thermal Stability

The temperature stability of the crude and purified enzyme extract was evaluated at different temperature values. For all assays, ca. 100 mg of enzyme extract was added to 50 mL buffer solution (50 mM sodium acetate, pH 5) and incubated at the selected temperature. Samples of 2 mL were withdrawn at time intervals from 5 min to 120 h for measuring residual enzyme activity according to Section 2.4.1. Residual activities were calculated by eq 1.

### 2.8 Modified Membrane Physical Characterizations

Unmodified (MF), modified (MM), and modified after filtration (MM_A_) membranes were dried for 48 h at 35 °C. The dry membranes were characterized by the following techniques:

1. Functional groups in a Fourier transform infrared spectrophotometer (ATR-FTIR; Vertex 70v, Bruker, Germany). 128 scans for analyses, 4 cm^–1^ spectral resolution over a range of 4000–400 cm^–1^;
2. Surface morphology in a scanning electron microscope (SEM, FEI Quanta 250, Eindhoven, Netherlands) with high vacuum (HV) 20 kV and a magnification of 2000 times, with prior metallization of a thin layer of gold at 50 mA for 130 s;
3. Contact angle by means of a goniometer (Contact Angle Micrometer, Tantec Inc., Schaumburg, IL), in which a microsyringe containing a drop of water of constant volume was used for analysis at three different points on the membrane surface;
4. ζ-potential: assessed for the modifying solutions prior to filtration (using solutions as prepared, without pH adjustments), enzymatic extracts, and membrane charge^55^ using a Beckman Coulter Delsa Nano Zeta Potential particle analyzer.

The mean pore radius (nm) was calculated using the Guerout–Elford–Ferry equation (eq 8) based on the water flow and porosity (eq 9) according to refs 56,57 . The thickness of the membranes was measured using the External Micrometer 100–125 mm Zaas 02.0006. The experiments were performed in triplicate, and the results are expressed as mean ± standard deviation.

8 $$r_{m}=\sqrt{\frac{\left(2.9-1.75\times \varepsilon \right)\times 8\times \eta \times l\times Q}{\varepsilon \times A\times \Delta P}}$$

where ε is the overall porosity of the membrane; η is the water viscosity (8.9 × 10^–4^ Pa·s^–1^); *l* is the membrane thickness (m); *Q* is the permeation rate (m^3^·s^–1^); *A* is the effective area of the membrane (m^2^); and Δ*P* is the operating pressure (Pa).

9 $$\varepsilon =\frac{W_{1}-W_{2}}{\rho \times A\times l}$$

where *W*_1_ is the weight of a wet membrane (kg), *W*_2_ is the weight of a dry membrane (kg), and ρ is water density (999 kg·m^–3^). To prepare the wet membranes, they were immersed in distilled water for 3 days, and after removing the excess water on the surface, they were weighed. The wet membranes were dried at room temperature (25 °C) for 7 days and then weighed.

All experiments in this work were performed in triplicate and with results expressed in average ± standard deviation.

## 3 Results and Discussion

### 3.1 Enzyme Extraction

The specific activity of lipase (in the powder seeds) was measured at 30 ± 4.1 U/mg of dry seeds. This indicates that extraction is crucial, as the specific activity of the seeds was 10 times lower than that of the crude extract, which measured 384.68 ± 27.23 U/mg dry seeds under the same conditions. This result suggests an increase in lipase production postgermination and extraction, as noted in refs 14,15 .^14,15^

Figure 1 shows the results of lipase extraction from defatted flaxseeds. Although the concentration of protein in the filtrate increased up to 24 h of extraction (reaching 520 ± 2.54 mg/L at maximum time tested), the specific activities in the suspension and filtrate peaked at 4 h (485 ± 46.01 U/mg protein). The decline of specific activities after this point can primarily be attributed to the extraction of nontarget proteins or contaminants. Additionally, some enzymatic inactivation may have occurred due to the extended extraction time,^58^ particularly if proteases were present in the contaminating proteins.^59,60^

**Figure 1:**
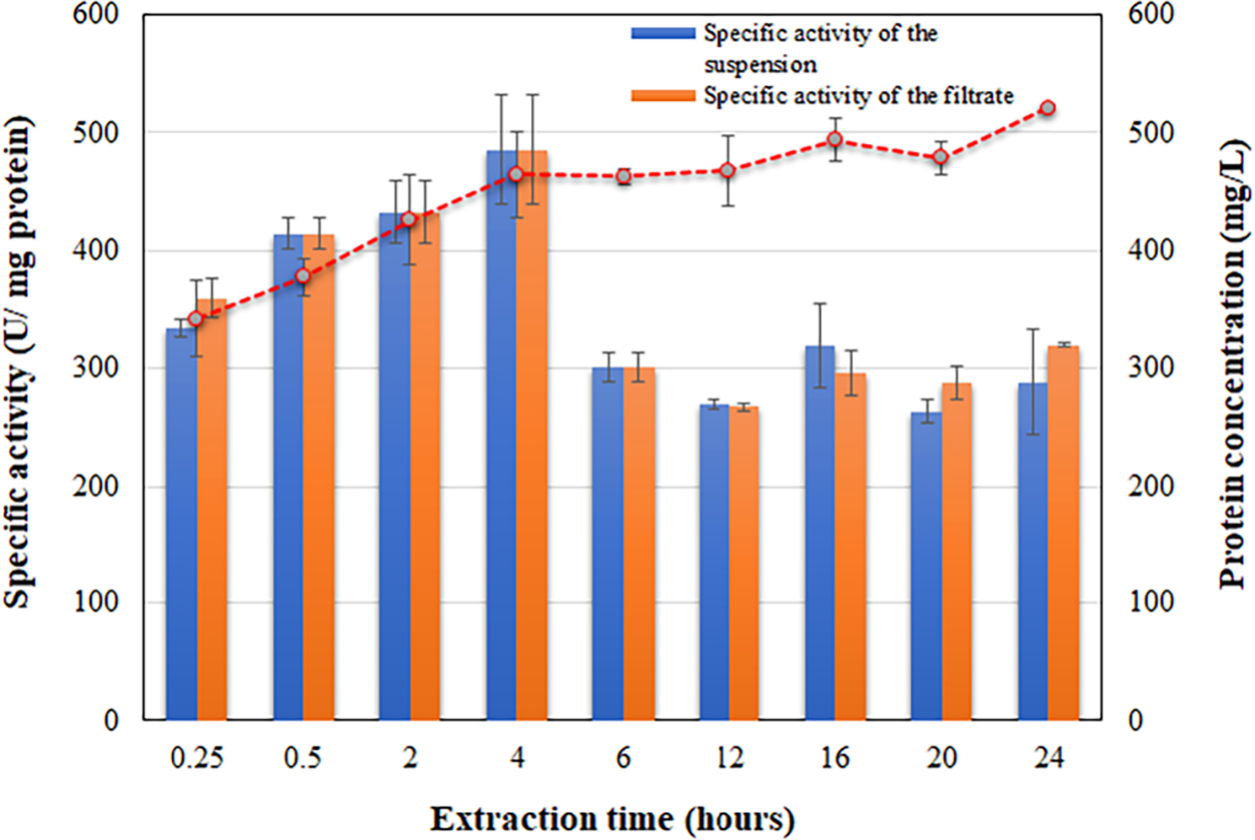
Extraction profiles of activity and protein over time for defatted flaxseeds using 100 mM sodium phosphate buffer, pH 7.0, as solvent, maintained in an ice bath. The mass of flaxseed powder to buffer volume ratio was 1:30. The dashed line represents the protein concentration in filtrate over time.

Lipase extraction using a buffer as a solvent (a physical technique) involves separating the enzyme from its biological matrix (the seeds) by disrupting the cellular structure and dissolving the enzyme in an aqueous buffer solution.^61^ The buffer not only stabilizes the pH but also provides sufficient ionic strength to facilitate the extraction process. Proteins such as lipase contain charged groups that interact with the surrounding medium. The buffer aids in dissolving lipases through mechanisms such as hydrogen bonding, ionic interactions, and the formation of a solvation layer around the enzyme, ensuring it remains in solution.^62^

Figure 2 illustrates that the lipolytic productivity of the dry flaxseed reached 6700 ± 88.20 U/g dry seeds after 4 h of extraction. Across a series of assays (14 in total) conducted on different days with various lots of seeds, productivities ranged from 6270 to 14970, yielding an average productivity of 11134.3 ± 2915.6 U/g dry seeds. This variation is acceptable, considering several factors involved in the lipase productivity, such as planting and harvesting conditions, oilseed variety, amount of germinated seeds^34,63^ and others.^64^ reported a lipolytic productivity of around 15 U (hydrolytic activity using olive oil a substrate) per gram of germinated *Jatropha curcas* L. seeds for 12 h extraction time at room temperature and in buffer at pH 6, highlighting a strong dependence of lipase activity on the amount of germinated seeds.

**Figure 2:**
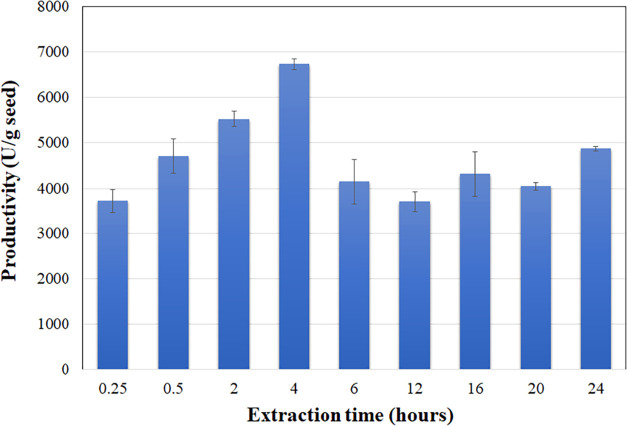
Productivity of lipolytic activity of defatted flaxseeds expressed as units per gram of dry seeds. Lipolytic activity was measured at 37 °C and pH 7.0, using olive oil as substrate.

### 3.2 Purification Filtration Experiments

#### 3.2.1 Enzyme Parameters

After the crude extract was obtained, the process involved sieving and subsequent centrifugation, leading to the formation of the preclarified extract. This extract then underwent various purification stages, including the use of commercial membranes, resulting in a clarified extract and eventually a purified extract. The purification parameters and outcomes are detailed in Table 1.

**Table 1:** Results of Specific Activity, Protein Content, Efficiency, and Purification Factor of Each Step of Lipase Purification (Sieving, Centrifugation, and Process with Membranes for Cycle)

step purification	protein content (mg/L)^a^	specific activity (U/mg protein)^a^	efficiency (%)^b^	purification factor^c^
crude enzymatic extract	497.90 ± 36.47	485.30 ± 46.01		
sieving	464.85 ± 59.27	615.05 ± 34.81	6.64	1.27
centrifugation	355.65 ± 45.46	686.34 ± 58.01	23.49	1.11
CN membrane	236.23 ± 86.25	3227.42 ± 115.39	33.58	4.70
modified PES membrane-permeated	132.19 ± 31.92	5612.01 ± 212.47	55.96	1.74
modified PES membrane-concentrated	206.23 ± 57.69	3306.04 ± 124.56	12.70^d^	1.02^d^

a Average ± standard deviation.

b (1 – Protein content obtained in step/previous protein content) × 100.

c Specific activity obtained in step/previous specific activity.

d The results for the concentrated fraction are related to CN membrane.

Through the purification steps, there was a significant reduction in protein content, decreasing approximately 3.8 times from 497.90 mg/L in the crude extract to 132.19 mg/L in the purified extract. Notably, the modified membranes exhibited the highest efficiency (≈56%) in achieving this reduction.

The specific activity exhibited a notable increase throughout the purification steps, rising from 485 U/mg in the crude extract to 5612 U/mg in the purified extract. Table 1 indicates a successful purification of the lipase with recovery observed in the permeate. All purification steps are essential for facilitating filtration through the modified membrane with reduced energy consumption, such as utilizing low pressures. Notably, the CN membrane step achieved the highest purification factor (4.70).

The purification process is significant in separating lipase from nontarget proteins, thereby enhancing its specific activity. Ultimately, the specific activity of the crude enzyme extract can be enhanced approximately 11.6 times through the purification process.

Analysis of the concentrated fraction after filtration with the modified membrane shows that not all of the enzyme is permeated, with lipase remaining in the concentrate. This can be attributed to factors such as the relationship between the size of the enzyme and the membrane pore as well as fouling on the membrane surface. Proteins can form a gel layer on the membrane surface, making filtration difficult and acting as an additional barrier to the filtration process.^13,33,42^

#### 3.2.2 Filtration Experiments in Modified Membrane

The performance of modified membranes was evaluated based on flux recovery rate (% FRR) and fouling: total (% *F*_T_), reversible (% *F*_R_), and irreversible (% *F*_I_) fouling are presented in Table 2.

**Table 2:** Fouling and Flow Recovery, Where Flow Recovery Rate (% FRR) and Fouling: Total (% *F*_T_); Reversible (% *F*_R_), and Irreversible (% *F*_I_) Fouling after Two Cycles of Filtration of the Clarified Enzymatic Extract

parameter data^a^	modified membrane
% FRR	74.38 ± 12.01
% *F*_T_	58.03 ± 7.29
% *F*_R_	32.41 ± 4.72
% *F*_I_	25.62 ± 12.01

a Average ± standard deviation.

As indicated in Table 2, there is a remarkable % FRR, signifying the substantial impact of the antifouling action proposed by GO^67^ on the modified membrane. Despite having a % *F*_T_ greater than 50%, % *F*_I_ is lower. This outcome is likely due to the retention of lipases and nontarget proteins on the membrane’s surface and within its pores, hindering greater permeation and leading to clogging. On the other hand, this suggests that, even after two cycles, the modified membrane remains reusable for additional cycles, as both rest washing and filtration washing can recover part of the protein content in the permeate. The specific activity attained in the purified state (permeated, Table 1) justifies the ongoing use of the modified membrane despite the observed fouling and flux values, with the membrane surface modification showing no interference with specific activity. Moreover, a significant portion of the fouling (32.41%) proves to be reversible, as evidenced by the results of buffer washes, contributing to the % FRR outcome.

Figure 3 shows the performance of the modified membrane during water filtration (*J*_0_ and *J*_3_) and two feed solution cycles (*J*_1_ and *J*_2_). *J*_0_ (39.22 ± 0.53 L·h^–1^·m^–2^) indicates that there was excellent flow stability, possibly due to the compaction phenomenon.^68^ This suggests that there is less possibility of membrane clogging, which can also be confirmed by the results presented in Table 2.

**Figure 3:**
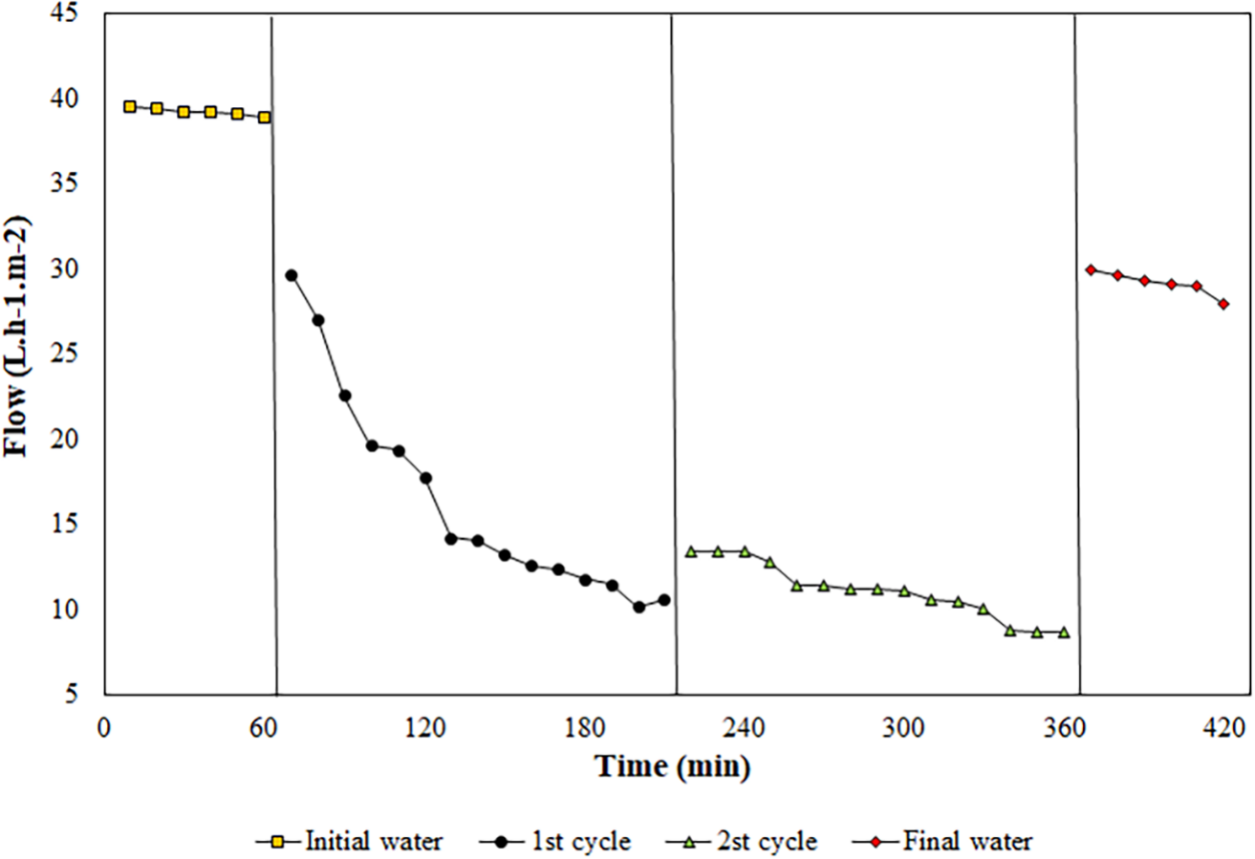
Water flows at the beginning of the process, feed solution (first and second cycles), and at the end of the process through the modified membrane over the filtration time (420 min).

The average flow corresponding to *J*_1_ was 16.46 ± 3.08 L·h^–1^·m^–2^. As seen in Figure 3, there was a large flow drop when the feed solution was filtered, indicating the interaction of the compounds with the membrane surface and pores. However, 97% of the lipase mass can be recovered after *J*_1_, as seen above. By filtering *J*_2_ (11.10 ± 1.17 L·h^–1^·m^–2^), there was an approximately 67% flow recovery. In the second cycle, there was a permeation of approximately 37% of the lipase mass, as well as in the first cycle. However, when the specific activity recovered was compared, this was low, only 1.6 times greater than that of the *J*_2_ feed solution (57.13 U/mg). On the other hand, when washing between *J*_2_ and *J*_3_, the buffer was able to recover 46.13% of the lipase mass with a specific activity of 2.45 times greater than that of the feed solution. However, 17.5% of the lipase mass was lost in the second cycle, although it showed approximately 72% of the specific activity of the feed solution. About the protein content, it was reduced by only 17.5 and 34.6% for the permeate and wash buffer solution, respectively, compared to the feed solution (329.93 mg/L). Finally, *J*_3_ (29.17 ± 5.11 L·h^–1^·m^–2^) showed excellent flow recovery (74.38%).

Other filtration cycles led to membrane clogging, possibly blocking proteins on the membrane surface, making permeation difficult. However, other ways of cleaning can be investigated in future works, like ultrasonic treatment,^69^ as well as higher operating pressure.^10^

### 3.3 Purified Lipase and Modified Membrane Physical Characterizations

As shown in Table 3, the insertion of GO nanoparticles on the membrane surface can impact both the reduction of the pore size and its hydrophilicity. In this way, permeability will also change.^65,68^ Permeability and the contact angle are inversely proportional. Based on this, there was a decrease in the permeability of the modified membrane (115.56 L·h^–1^·m^–2^·bar^–1^) compared to the pure membrane (19451.00 ± 119.33 L·h^–1^·m^–2^·bar^–1^). This reduction can be attributed to the higher contact angle of the modified membrane (93.83°) compared to the pure one (60.00 ± 2.16°).

**Table 3:** Membrane Modification: Characterization and Evaluation Parameters

samples	permeability (L·h^–1^·m^–2^·bar^–1^)	ζ-potential^a^	membrane surface charge	pore radius	contact angle (°)
PEI		+5.57 mV	–14.33 mV		
GO-TA		–35.60 mV	–10.27 mV		
membrane pure			–13.15 mV	56 nm	60.00 ± 2.16
membrane modified	115.56 ± 16.59		0 mV	4.34 nm	93.83 ± 9.60

a Aqueous solution of modifying compounds.

These results suggest that GO nanoparticles reduced the effective pore size, as expected, and thus modified the microporous structure of the membrane, reducing water permeation.^65^ On the other hand, after filtering the feed solution, the contact angle was reduced (85.00 ± 12.91 L·h^–1^·m^–2^·bar^–1^), becoming more hydrophilic. It is suggested that there was an increase in membrane hydrophilicity when the feed solution encountered the PEI layer, improving its surface dispersion, and consequently maintaining enzyme activity.^66,27^

The modified membrane exhibited a smaller average pore radius of 4.34 nm, compared to 56 nm for the unmodified one, as shown in Table 3. This radius disparity stems from differences in membrane thickness and porosity: the modified membrane exhibited a thickness of 100 μm and a porosity of 1.0, while the unmodified membrane measured 120 μm in thickness with a porosity of 0.84. These results further confirm that coating the membrane surface with TA-functionalized GO nanoparticles decreases porosity, permeation, and flow area, thereby enhancing the selectivity of the modified membrane.^27,38^

The codeposition of PEI on the pure PES membrane retained a negative surface charge, despite the inherent positive charge of the PEI aqueous solution, as can be seen in Table 3. This is likely attributable to the limited presence of positive cations (NH_4_^+^) dispersed in the PEI solution. Consequently, the carboxyl groups within the PES structure segregated to the membrane surface, sustaining the negative charge on the membrane.^75^ Otherwise, the carboxyl groups in the GO suspension impart a significant negative charge, thereby diminishing PEI codeposition while preserving the negative surface charge of the membrane. Meanwhile, the inherent positive charge of TA induces electrostatic interactions and hydrogen bonds with PEI, fostering robust adhesion between the layers.^76^

The membrane surface was modified using tannic acid, graphene oxide, and polyethylenimine (PEI), whose strong hydrogen bonding and electrostatic interactions enable the formation of a stable and multifunctional coating. This modification was carried out to improve the hydrophilicity, antifouling properties, and mechanical stability of the membrane, as well as to enhance its affinity toward biomolecules such as lipases.^23–31^ The effectiveness of this modification can be seen in the experimental results, which indicate significant improvements in membrane performance and enzyme recovery. In addition to our findings, several studies in the literature have also reported the positive impact of such surface modifications on enhancing membrane selectivity, permeability, and stability, further reinforcing the effectiveness of this approach.^77,78^

The final deposition layer exhibited a neutral charge, suggesting that the numerous amino groups in PEI neutralized the negative charge of TA-GO.^76^ Considering the negative charge of the clarified extract (−10.20 mV), it is proposed that a portion of the lipase managed to traverse the negative layer (TA-GO) without encountering electrostatic repulsion. In contrast, the phosphate buffer successfully recovered lipase from the filtrate pores, exerting a force greater than the repulsion force of the negative layers.^79^ The schematic of the modified membrane and possible interactions with lipase and other compounds can be seen in Figure 4A.

**Figure 4:**
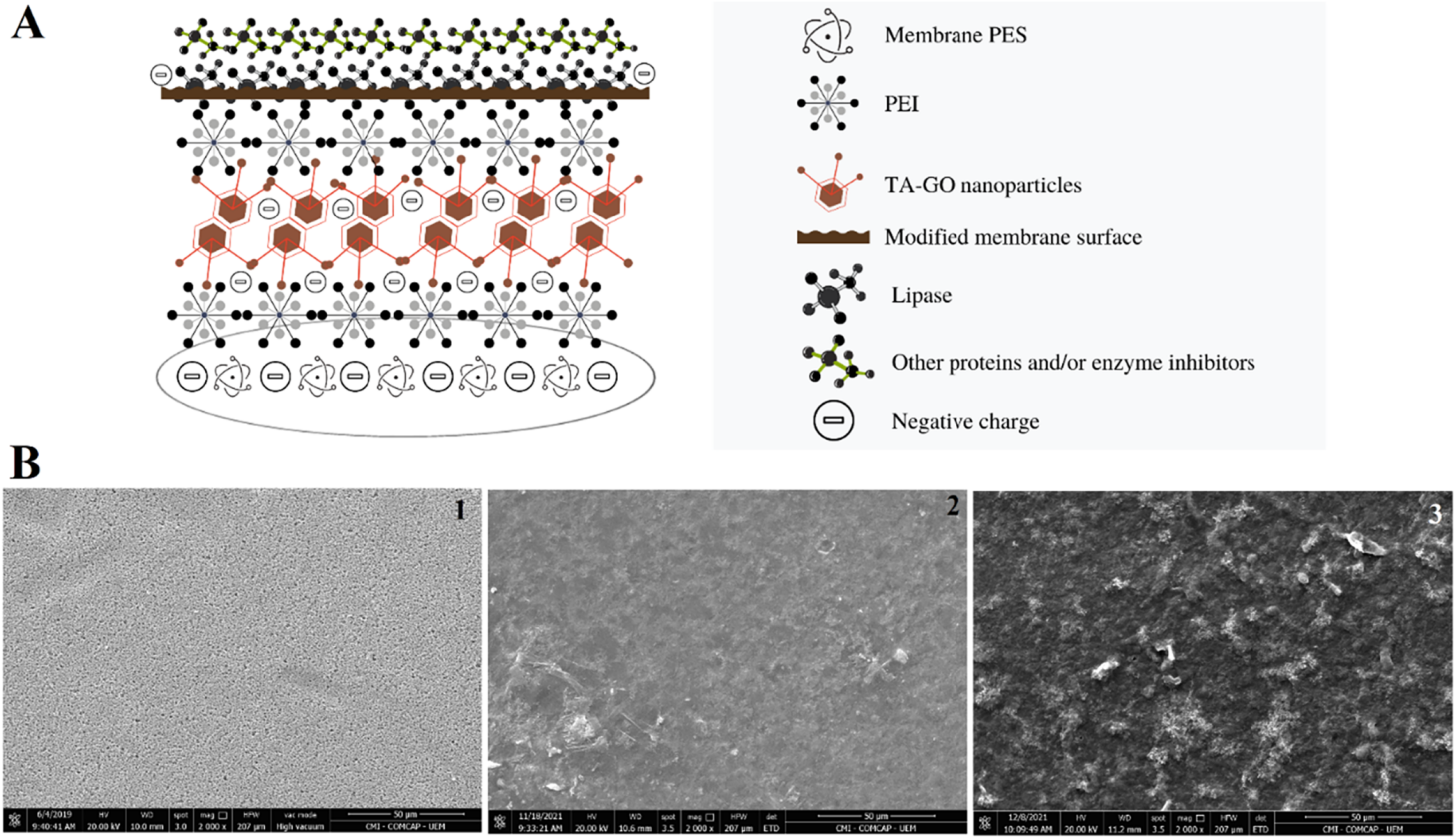
(A) Interactions between the modified membrane layers and the feed solution (clarified extract) based on ζ-potential and surface charge analysis. (B) SEM morphology of the pure membrane (1), modified (2), and after filtration of the feed solution (3) with a magnitude of 2000 times.

The membrane morphologies were evaluated by SEM micrographs (Figure 4B) at a magnification of 2000 times. In Figure 4B-1, pores can be seen across the entire surface of the sheer membrane, which changed after the modifications (Figure 4B-2). The surface of the modified membrane is rough, characteristic of graphene oxide, also observed in membranes containing TA-GO, and is attributed to the polymerization reaction between them.^65^ Some whitish spots that may be related to PEI are also observed.^52^ In Figure 4B-3 (SEM image of the modified membrane after the filtration experiment), it is possible to relate the new whitish rough appearance to the impregnation of lipase on the membrane surface.

Membrane characterization was performed to confirm the change in the membrane surface. Figure 5A shows the functional groups obtained by ATR-FTIR analysis for the unmodified membrane and modified membrane and after the second filtration of the feed solution. The spectrum of the unmodified membrane agrees with those previously reported in the scientific literature. Peaks below 750 cm^–1^ can be attributed to the C–S bond, since its molecular mass is greater than the other bonds and it has a low vibration frequency. At 834 and 1011 cm^–1^ refer to the C–H aromatic rings deformation of the substituted ring; between 1050 and 1250 cm^–1^ to the characteristic O=S=O bond, standing out to the peaks related to the C–O and C–O–C bonds at 1235 cm^–1^ for the aromatic ether; 1100–1300 cm^–1^ for C–O bonds; 1450–1500 cm^–1^ C–C bonds; 1500–1750 cm^–1^ to C=C bonds; and finally, 3250–3000 cm^–1^ for the C–H bonds.^52,65,80^

**Figure 5:**
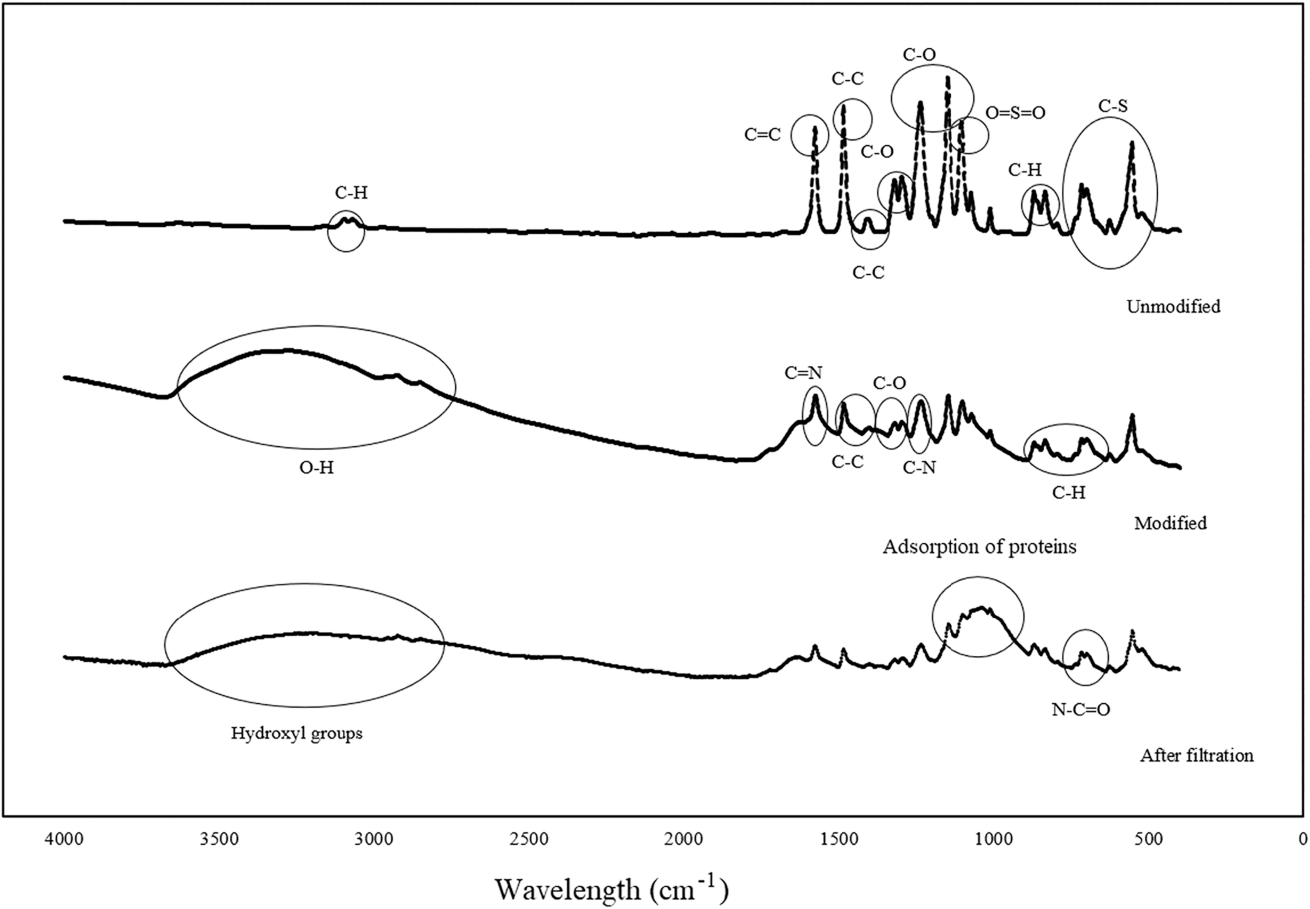
FTIR-ATR spectra showing functional groups in the unmodified, the membrane modified with PEI and TA-GO, and the membrane modified after second filtration of the feed solution, presented as wavelength data (cm^–1^).

The modified membrane showed changes in the intensities of several bands compared with the unmodified membrane spectrum. The polymeric layer formed by PEI can be observed by the bands in the region 1280–1180 and 1600 cm^–1^, related to C–N and C=N bonds, respectively. The following peaks can be referred to the structure formed by TA-GO: 1300–1100 cm^–1^ (C–O), 900–690 cm^–1^ (C–H) derived from the aromatic ring (C–C at 1580 and 1450 cm^–1^). The region 3600–3000 cm^–1^ (O–H) had an increase, possibly due to the carboxylic acid present in the PEI and TA-GO chemical structure.^81,82^

In the spectrum after filtration of the clarified extract, a peak close to 617 cm^–1^ is observed, which can be attributed to the N–C=O bond of amides present in the lipase structure.^83^ Furthermore, the modification in the 2400–3400 cm^–1^ bands suggests the presence of hydroxyl groups that are in the aqueous phosphate buffer solution with lipase. Likewise, the change of bands in the 800–1200 cm^–1^ region suggests the adsorption of enzymes on the membrane surface.^84^

### 3.4 Influence of pH and Temperature on the Hydrolytic Activity of Flaxseed Lipase

Figure 6 shows the hydrolytic activity profiles of flaxseed lipases as functions of pH (Figure 6A) and temperature (Figure 6B). Notably, the solubilization process did not modify the catalytic properties of flaxseed lipases, at least within the pH and temperature ranges showcasing their maximum hydrolytic activity. Both lipases in the crude extract and those in the purified extract exhibited similar behavior in relation to pH (5), which is considered the optimum pH. This acidity aligns with the nature of flaxseed lipases, resembling the acidic traits of castor bean and lupine lipases as documented in refs 13,70 . Moreover, the acidic character of flaxseed lipases, with an optimal pH of 4.5, has been previously reported in ref 38. Regarding temperature, the lipase in the crude extract demonstrated increased activity at 25 °C. In contrast, the purified extract exhibited optimal catalytic activity in the temperature range of 37–45 °C. However, the hydrolytic activity for both is still high over a wide range of temperatures. Similar findings regarding higher activity in the temperature range of 30–40 °C have been previously reported for several seed lipases, such as lipases from African bean seeds, French bean seeds, castor seeds, rapeseed, Barbados nut, French peanuts, wheat seed, and coconut seed.^13^

**Figure 6:**
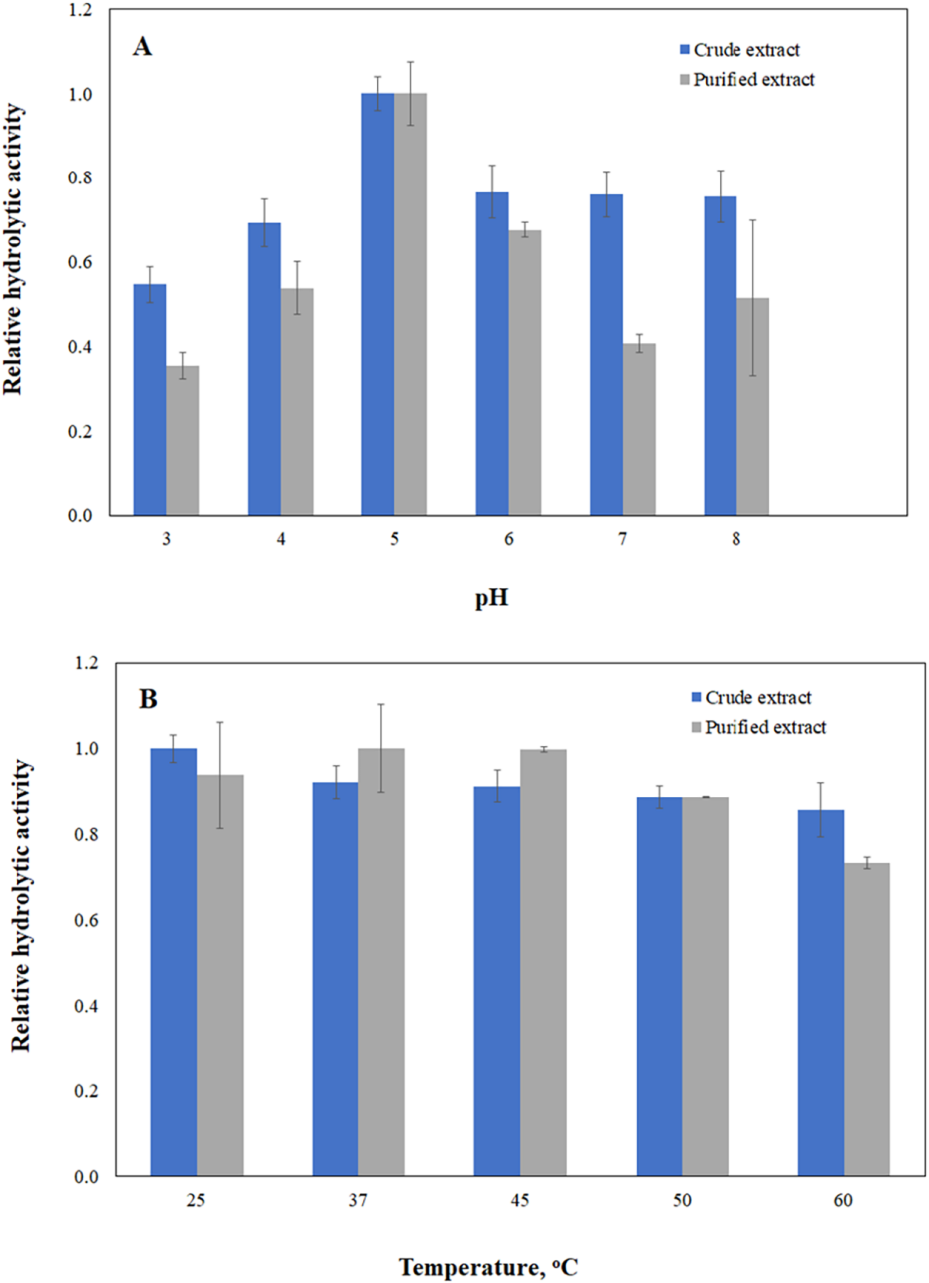
Profiles of hydrolytic activities of flaxseed lipases as a function of (A) pH at 37 °C and (B) temperature at pH 7.0. Substrate: emulsified olive oil.

Figure 7 shows the results of stability tests at different temperatures and pH values of flaxseed lipases (crude extract and purified extract). The crude extract exhibited high stability at almost all pH and temperatures tested, retaining more than 80% of the initial activity after 4 h of incubation. In contrast, the purified extract demonstrated heightened stability, specifically notable at pH 5 and within the temperature range of 37–45 °C. In both instances, it retained over 95% of the initial activity after a 4 h incubation period. These results show that lipolytic activity was preserved at acidic pH in the presence of a substrate (emulsified olive oil). Regarding thermostability, flaxseed lipase becomes attractive for several industrial applications,^71^ mainly in modifications of oils and fats, where higher temperatures favor better mass transfer due to the reduction in the viscosity of the medium.^72,73^ Furthermore, higher operating temperatures of a bioreactor could impede microbial growth, particularly important for food applications.^74^

**Figure 7:**
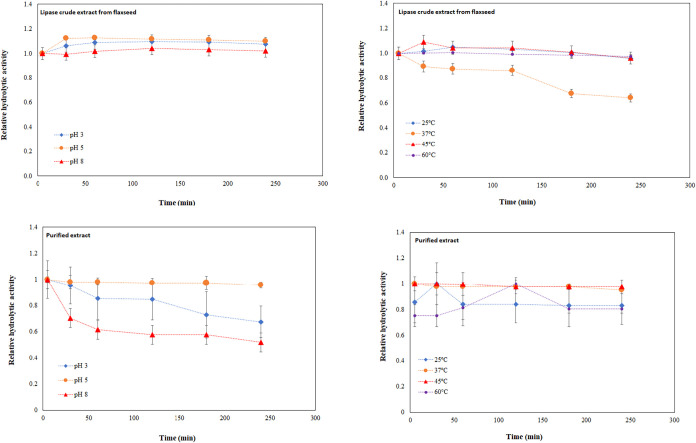
Stability assays at 37 °C and pH range of 3–8, and pH 5 at a temperature range of 25–60 °C. Activities were measured using emulsified olive oil as substrate, and the initial activity was taken as 100%.

Following purification, denaturing SDS-PAGE (Figure 8) revealed a single lipase with an apparent molecular weight of 32.4 kDa, produced under the conditions utilized in this study. Similar molecular sizes for plant-derived lipases have been reported in previous studies, ranging from 9.4 to 143 kDa in ref 13 and from 39.2 to 52.9 kDa in ref 2.^2^

**Figure 8:**
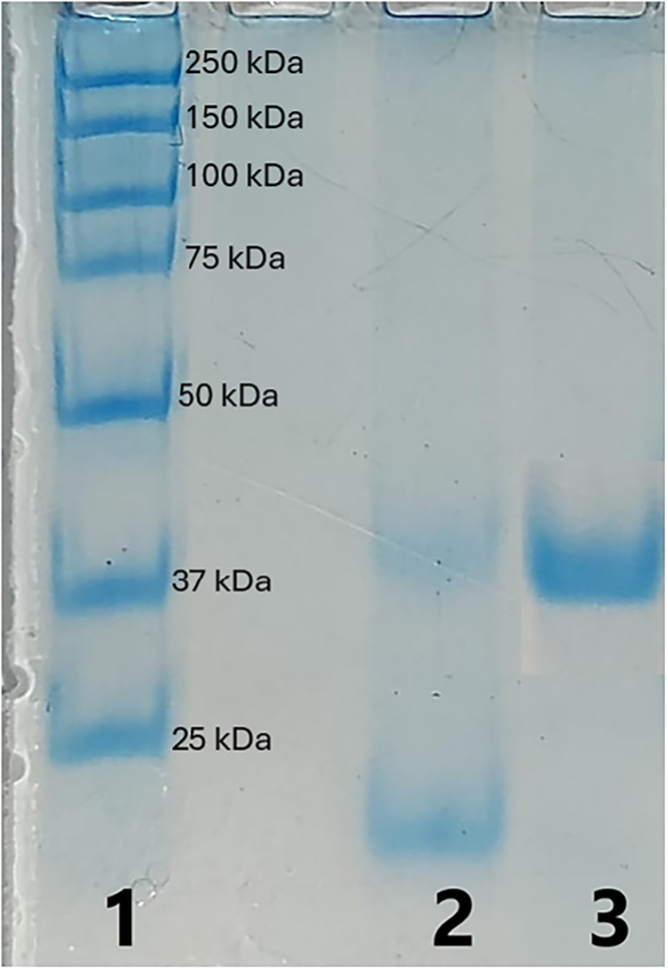
SDS-PAGE electrophoresis of flaxseed lipase. Lane 1—molecular weight standards; Lane 2—crude extract samples; and Lane 3—purified extract sample.

Although the results obtained demonstrate the potential of the proposed approach, certain limitations and challenges must be considered for its broader application. The membrane system showed satisfactory efficiency in lipase purification and could be reused; however, a gradual decrease in permeate flux after successive cycles indicates the need to improve regeneration and cleaning strategies to maintain the performance over time. The transition from bench-scale experiments to industrial implementation also poses difficulties, mainly related to the control of fouling, the uniform deposition of modifying agents, and the overall economic feasibility of large-scale membrane functionalization. Moreover, variations in the biochemical characteristics of lipases from different seed sources emphasize the importance of further investigating the enzyme diversity and extraction parameters.

Future work should aim to refine membrane surface engineering through alternative nanomaterial combinations, enhance operational stability under continuous use, and integrate this purification step with enzyme immobilization or catalytic systems. Advancing these aspects will contribute to the development of efficient, durable, and environmentally sustainable membrane-based technologies for enzyme purification and biotechnological applications.

Ongoing research is essential in the biotechnology sector to identify new seed lipase. Depending on their source, these lipases may have diverse biochemical properties that could partially replace microbial lipases in various industrial applications. An acid lipase was identified, demonstrating favorable catalytic properties for food chemistry applications.

A key highlight of this study is the use of membrane-based purification as an effective strategy for enzyme isolation. Functionalized graphene oxide nanoparticles with tannic acid, coated on a poly­(ether sulfone) membrane alongside polyethylenimine, were characterized. This modified membrane effectively purifies lipase, resulting in an approximately 11.6-fold increase in the specific activity. The presence and characteristics of lipase, along with its functional groups and specific activity, indicate that membrane modification preserves its conformational structure. Notably, the modified membrane demonstrates reusability across two filtration cycles, with a 74.38% flow recovery rate, showcasing the antifouling effect proposed by tannic acid-functionalized graphene oxide.

These findings underscore the potential of engineered membranes as powerful, reusable platforms for efficient enzyme purification, setting the stage for future studies focused on optimizing reaction conditions and exploring broader catalytic applications for this promising lipase.
